# Opening Editorial: CPD and Lifelong Learning: a call for an evidence based discussion

**DOI:** 10.15694/mep.2019.000001.1

**Published:** 2019-01-04

**Authors:** Samar Aboulsoud, Helena Filipe

**Affiliations:** 1 Qatar Council for Healthcare Practitioners; 2 Hospital das Forças Armadas/PL-EMGFA and Hospital SAMS

**Keywords:** Continuing Medical Education, CME, Continuous Professional Development, CPD, Substantial Equivalency, Inter-professional Education, IPE

## Abstract

This article was migrated. The article was marked as recommended.

Globalization of Continuing Professional Development (CPD) is driven by shared educational principles and management trends that facilitate international standards in CPD. These are enforced through instructional design, clinical teaching and assessment methods based on education theory, effective communication skills, and CPD systems strengthening medical education global principles.

The growing interest in recognizing CPD and in globalizing CPD accreditation standards has prompted several collaborative international initiatives which include the promotion of national CPD accreditation systems, international collaborations and partnerships, publications of research data and the mutual recognition of international CPD systems and programs.

## Introduction

In 2017 AMEE established a new committee for Continuing Professional Development (CPD). The committee serves as the voice of AMEE in the global CPD community and provides opportunities to emphasise the importance of CPD in the medical education continuum by engaging the several stakeholders and by facilitating collaborative CPD initiatives across the globe.

Fundamental CPD principles, as embraced by the committee, reflect a borderless CPD and a substantial equivalency between CPD accreditation systems ( Filipe et al., 2018). Aiming to vividly translate these principles into practice, we are welcoming CPD themed publications from all over the globe and encourage the CPD community to share their success stories and address their challenges in the CPD themed issue. This themed edition is planned to be a venue to promote exchange of ideas and stimulate innovation.

## CPD without borders

With the ever-growing cross-country mobility of both patients and health care professionals, ensuring a safe and high quality patient care has become increasingly essential. Professional migration and facilitated mutual recognition of qualifications however, make us question the fitness of the current status of reciprocated agreements in CME/CPD among countries for safeguarding patient care and maintain international accountability of healthcare professionals ( Filipe, et al. 2014). Congruency between the fundamental principles and outcomes of CME/CPD frameworks and accreditation systems would thus be valuable. Healthcare professionals should have their CPD recognized by local, national and international organizations requiring CME for maintenance of competence purposes to ensure patient safety and enhance transparency and professional accountability.

## Substantial equivalency

CPD accreditation systems should ensure that learning activities are of good educational quality and based on sound educational principles.

There has been growing interest concerning the concept of substantial equivalency between CPD accreditation systems. Cultural differences, as well as specific regional healthcare needs within different healthcare systems, should not be neglected. Therefore, a unified system should not be our goal. Besides being impractical and difficult to achieve, it might also limit innovations in accreditation of CPD systems and programs ( McMahon et al., 2016). It is more realistic, however, for different systems across the globe to agree on a conceptual foundational framework of accreditation with common sets of principles, definitions and essential characteristics of the accreditation standards. Full harmonisation of accreditation systems is neither possible nor desirable. Instead, shared principles and values with substantial commonality should be encouraged. As international systems vary in detail, systems’ differences would also be expected and accepted.

## CME/CPD in the medical education continuum

There is a global move to integrate CME and CPD as a fundamental part in the medical education continuum. There are marked variations in CPD across the world also when compared to undergraduate and postgraduate medical education. Variations include the most often-unstructured non-competence based activities and programs compared to the fully structured outcome based curriculum in undergraduate and postgraduate medical education ( Sherman, 2018). There are also complexities in CPD being linked to recertification and maintenance of license to practice. Multiple stakeholders with variable sometimes-conflicting agenda add to the complexity of the process.

This CPD themed issue is an attempt to document variations and trends across the world in relation to CPD frameworks, and accreditation /revalidation systems. Given the professional, national and regional variations of CPD systems, we hope this edition will incite reflection, research and discussion among the CPD community: CPD educators and providers, healthcare professionals and other CPD stakeholders ( Filipe at al., 2016). Systems based CPD and competency based continuing education, educational theory applied in practice, educational and clinical outcomes overarching, CPD scholarship and research, professional accountability, CPD value creation in healthcare systems, CPD organizational leadership, CPD international agreement, all are exciting topics to further insight within the realm of CPD.

## Leadership in CPD

**Figure 1.**
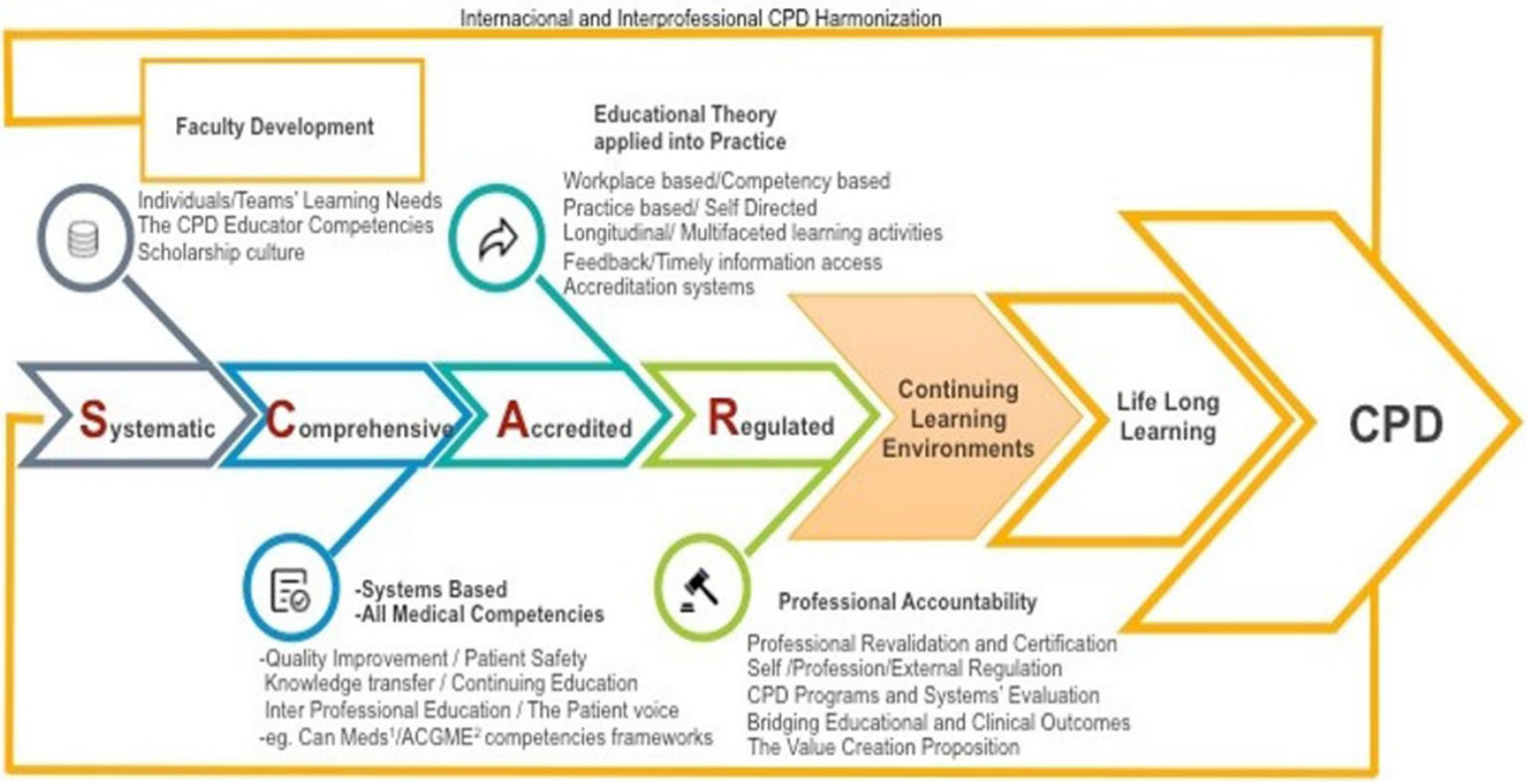
Leadership in CPD

Four foundational principles are highlighted in ( Figure 1) as the backbone of effective CPD: it should be systematic, comprehensive, accredited and regulated (SCAR) ( Filipe et al., 2018). Each of these principles can abridge several CPD topics unlocking exciting pathways to explore.

As systematic, effective CPD should incorporate the CPD learning cycle focusing the practitioner and healthcare teams educational needs in a continuous iterative revision and improvement, calling for the continuing enhancement of the CPD educator’s competencies and fostering a scholarship culture in a continuing effort of faculty development ( Filipe, 2014).

As comprehensive, CPD should be seen as a cornerstone of healthcare systems at the clinical, organizational and public health levels. It should include all medical competencies as encompassed in the CanMeds ( Frank J.R. et al., 2015) or ACGME ( Holmboe, E.S. et al, 2016) physician competencies frameworks. CPD programs should embed quality improvement, patient safety and knowledge transfer. The patient contribution and interprofessional education by design should meaningfully make part of the CPD curricula.

Accreditation conceptually encloses the application of educational theory into practice and include workplace outcome based education and self-directed learning, longitudinal, multifaceted learning experiences with effective feedback. Accreditation systems play an important educational role in CPD.

Professional accountability relies in clearly demonstrating CPD through documentation of self-reflection and external evaluation with or without credit award. The CPD educator is increasingly challenged to bridge educational to clinical outcomes, which in turn increases value creation of CPD and thus professional accountability.

Leaders of healthcare organizations and authorities, societies and colleges and policy makers should facilitate effective continuing learning environments as an imperative to nurture a culture of lifelong learning, which lies at the core of CPD ( Davis & McMahon, 2018).

## A call for an evidence based discussion

**Figure 2.**
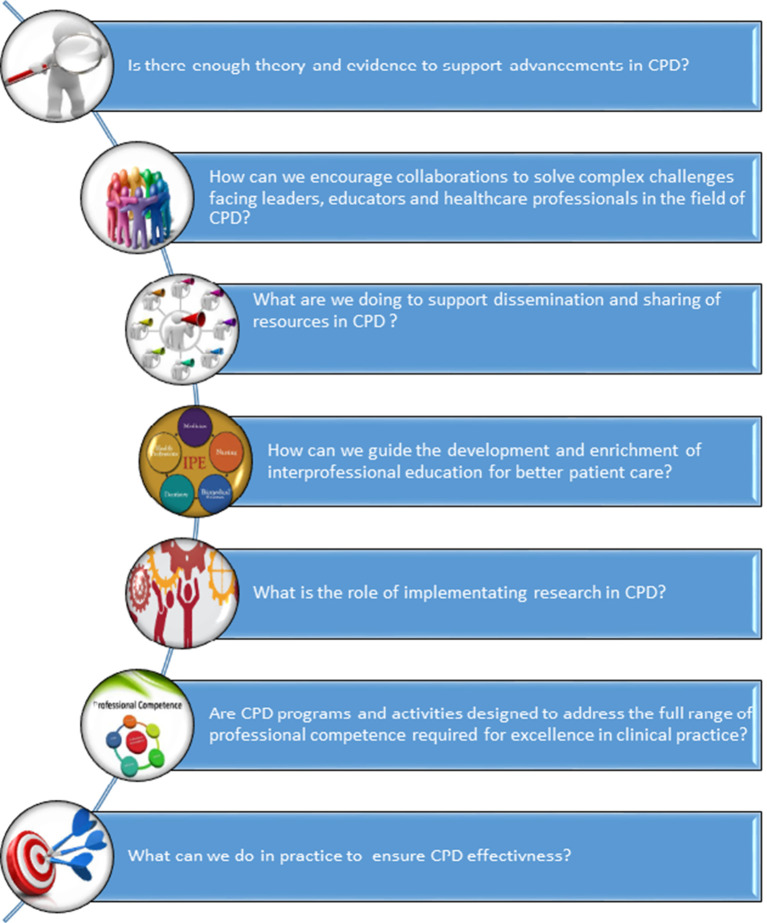
Several key questions in CPD

**Figure 3.**
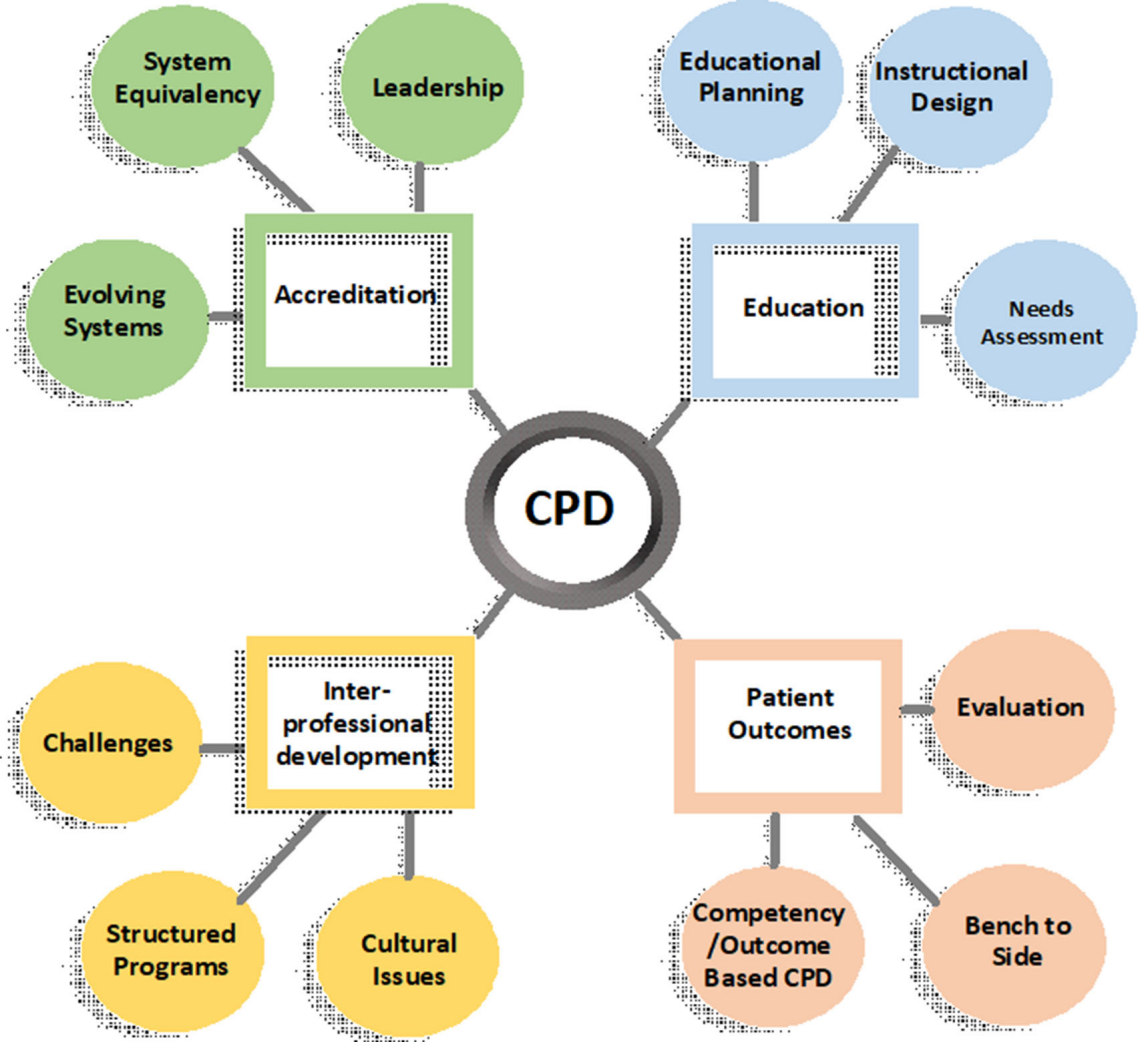
Mind map with some topics for exploration within CPD

In this opening editorial for our CPD themed issue, amongst others we encourage authors to consider several key questions ( Figure 2) and several concatenate concepts and ideas ( Figure 3).

If it is fascinating to study how the healthcare workforce continuing education and professional development has been progressing, it is even more tempting to glimpse into the future of CPD. Several key thinkers in the field have already offered us some rich food for thought on the next future challenges that the CPD educator is already facing in their educational practice (Dave Davis, 2018 and Sargent et al., 2018).

## Conclusion

We hope this themed edition will provide a platform for exchanging experience, thoughts and ideas towards harmonization, albeit observing customization, to meet regional specific healthcare needs, showcase educational innovations and envision future trends in CPD.

## Take Home Messages

• Mutual recognition in fundamental principles and outcomes of CME/CPD frameworks and accreditation systems would be valuable to ensure patient safety and quality of health care.
• There is a global move to integrate CME and CPD as a fundamental part in the medical education continuum.
• Effective CPD should be systematic, comprehensive, accredited and regulated (SCAR).
• Continuing learning environments should be facilitated by those in healthcare leadership positions to nurture a culture of lifelong learning, the heart of CPD.

## Notes On Contributors

**Dr. Samar Aboulsoud, MBBCH, MSc int. med, PhD, MSc Med Ed, FHEA, MAcadMEd** currently serves as the Acting Chief Executive Officer of the Qatar Council for Healthcare Practitioners. She is also the Director for Accreditation and Health Profession Education Department. She was the thought leader and project manager for the establishment of the national CME/CPD accreditation system for the state of Qatar.She is the Chair of Qatar CPD Accreditation Committee. She is an Associate Professor of Internal Medicine in both Cairo and Qatar University Schools of Medicine. She was selected one of the top 100 CEO’s in the Arab world for the year 2017. She is a fellow of the Higher Education Academy of Medical Educators in the UK and the International Academy for CPD Accreditation. Her research interests are medical education and clinical guidelines.

**Helena Filipe, MD, MSc-Medical Education, FSACME, AFAMEE** is a Consultant of Ophthalmology at Hospital das Forças Armadas/PL-EMGFA and Hospital SAMS, Lisbon, Portugal. She currently serves the International Council of Ophthalmology (ICO) as chair of the CPD area of focus, serves the Association for Medical Education in Europe (AMEE) CPD Committee as co-chair, the Association for Research in Vision and Ophthalmology (ARVO) CME Committee, the Global Alliance for Medical Education (GAME) Education Committee and the Board of the Portuguese Medical Association College of Ophthalmology. She is an invited collaborator of the Department of Medical Education of the Faculty of Medicine of the University of Lisbon.

## Declarations

The author has declared the conflicts of interest below.

Drs Samar Aboulsoud and Helena Prior Filipe are guest Theme Editors for the theme of Continuing Professional Development in AMEE MedEdPublish.

## Ethics Statement

Not required for this opening editorial.

## External Funding

This article has not had any External Funding
