# Supplementary material for: Opening Editorial: CPD and Lifelong Learning: a call for an evidence based discussion
Source: MedEdPublish (2016). 2019 Jan 4;8:1. [Version 1] doi: 10.15694/mep.2019.000001.1 (PMC10711967; doi:10.15694/mep.2019.000001.1)
Supplement: Peer review history

# Reviewer report: Reviewer response for version 1

**DOI:** 10.21956/mep.19748.r29619

**Published:** 2019-01-20

**Authors:** Leila Niemi-Murola

**Affiliations:** 1 University of Helsinki

This review has been migrated. The reviewer awarded 5 stars out of 5 Thank you for the invitation to review this interesting commentary. I absolutely agree about the need to harmonize the concept of CPD/CME. In some European countries there is mandatory CPD with incentives and in other countries it is voluntary although strongly recommended. Harmonization of the CPD would enhance the attractiveness of international congresses. We can assume that CPD will be mandatory in all EU countries sooner or later.I absolutely agree with the authors about the SCAR as the backbone of CPD (systematic, comprehensive, accredited and regulated). I liked the system and the illustrative figures presented by the authors. Most CPD activities have very traditional design. Focus is often on sharing knowledge. However, we are shifting towards competence-based medical education. This should be implemented in CPD as well as undergraduate and specialist education.

Reviewer Expertise:

NA

No decision status is available

# Reviewer report: Reviewer response for version 1

**DOI:** 10.21956/mep.19748.r29620

**Published:** 2019-01-14

**Authors:** David Bruce

**Affiliations:** 1 None

This review has been migrated. The reviewer awarded 5 stars out of 5 I welcome this editorial which highlights the need for sharing what is understood and practiced as Continuing Professional Development across the globe. The call for an evidence-based discussion is timely and important as the focus on the safety and quality of patient care and regulation of medical practitioners drives forward.While the clearly defined outcomes and curricula of undergraduate and postgraduate training has enabled sharing of content, methods and standards across Medical Schools and Postgraduate training organisations, the lack of any formal CPD curriculum (which there should not be) has perhaps hindered sharing of what works best under what circumstances. This editorial helpfully points to the SCAR CPD principles that all can apply. It asks a series of questions for educationalists to debate share experiences and for researchers to explore further. The SCAR principles appear straightforward and form the authors’ backbone of effective CPD – but may have challenges for some systems. Whether all CPD comprehensively covers all areas of each individuals practice including their ability to work in multidisciplinary teams, and variations in what is being accredited (the providers, the events or even individual learning) across the globe will be interesting to hear. This gives plenty scope for sharing and future discussions.Future challenges and directions for CPD are touched on, but what should also be considered is how we best support individual practitioners who are at risk of poor performance. While CPD enables practitioners to provide high standards of care for their patients, a medical career is long and arduous, and illness, life events and just losing interest can affect an individual’s performance. It would therefore also be helpful to consider what needs to be in place to pick up early signs of poor performance such as disengagement and diminished insight. We can consider what leadership is required to create educational environments that help and support such individuals back to providing quality care. Like all good editorials this piece gives us food for thought and a call to share our experiences, research and planned future developments. I recommend this editorial to all educationalists and hope that many take up the challenge to share their experiences and expertise.

Reviewer Expertise:

NA

No decision status is available

# Reviewer report: Reviewer response for version 1

**DOI:** 10.21956/mep.19748.r29622

**Published:** 2019-01-13

**Authors:** Susmita Reddy Karri

**Affiliations:** 1 Topiwala national medical college and BYL nair charitable hospital, mumbai. india

This review has been migrated. The reviewer awarded 5 stars out of 5 A very comprehensive paper and a good initiative by AMEE. The very thought of CPD without borders, but at the same time, not ignoring the local cultural needs looks very fresh. A good read.

Reviewer Expertise:

NA

No decision status is available

# Reviewer report: Reviewer response for version 1

**DOI:** 10.21956/mep.19748.r29623

**Published:** 2019-01-05

**Authors:** Balakrishnan Nair

**Affiliations:** 1 Centre for Medical Professional Development and University of Newcastle

This review has been migrated. The reviewer awarded 5 stars out of 5 This well written and referenced editorial discusses the need for “CPD without borders” without neglecting the local cultural and health care needs. CPD is an area which will need more evidence base including positive patient and community outcomes. The CPD concepts and areas to explore are well articulated in the figures. I was wondering SCAR should have another A, Acceptability to it, both from the providers and recipients?I enjoyed reading reading this editorial.

Reviewer Expertise:

NA

No decision status is available

# Reviewer report: Reviewer response for version 1

**DOI:** 10.21956/mep.19748.r29621

**Published:** 2019-01-04

**Authors:** Sateesh Babu Arja

**Affiliations:** 1 Avalon University School of Medicine

This review has been migrated. The reviewer awarded 5 stars out of 5 I would like to thank the authors for presenting an important topic CPD in medical education. I read this editorial with much interest and enjoyed reading it. Even though there is some overlap of the concepts of CPD and CME, they differ. CME focuses on the activities which enhance competencies or skills required to practice as a physician. Whereas CPD focuses on activities which enhance broad competencies or skills including patient care, teaching, research, assessment, administrative, and leadership. The editorial is written very well, very informative, and comprehensive. It is interesting to see AMEE took the initiative and formed the committee for continuing professional development. I liked the figures presented and they made understanding easy. I recommend this editorial for faculty members and the leadership who oversee faculty development activities. Thank you.

Reviewer Expertise:

NA

No decision status is available
